# The Arabinogalactan Protein Family of *Centaurium erythraea* Rafn

**DOI:** 10.3390/plants10091870

**Published:** 2021-09-09

**Authors:** Danijela M. Paunović, Katarina B. Ćuković, Milica D. Bogdanović, Slađana I. Todorović, Milana M. Trifunović-Momčilov, Angelina R. Subotić, Ana D. Simonović, Milan B. Dragićević

**Affiliations:** Department of Plant Physiology, Institute for Biological Research “Siniša Stanković”—National Institute of Republic of Serbia, University of Belgrade, 11060 Belgrade, Serbia; katarina.cukovic@ibiss.bg.ac.rs (K.B.Ć.); milica.bogdanovic@ibiss.bg.ac.rs (M.D.B.); slatod@ibiss.bg.ac.rs (S.I.T.); milanag@ibiss.bg.ac.rs (M.M.T.-M.); heroina@ibiss.bg.ac.rs (A.R.S.); ana.simonovic@ibiss.bg.ac.rs (A.D.S.); mdragicevic@ibiss.bg.ac.rs (M.B.D.)

**Keywords:** arabinogalactan proteins, FLAs, gene expression, hydroxyproline-rich glycoproteins, MAAB classes, mechanical wounding, organogenesis, protein kinases, ragp, somatic embryogenesis

## Abstract

*Centaurium erythraea* (centaury) is a medicinal plant with exceptional developmental plasticity in vitro and vigorous, often spontaneous, regeneration via shoot organogenesis and somatic embryogenesis, during which arabinogalactan proteins (AGPs) play an important role. AGPs are highly glycosylated proteins belonging to the super family of O-glycosylated plant cell surface hydroxyproline-rich glycoproteins (HRGPs). HRGPs/AGPs are intrinsically disordered and not well conserved, making their homology-based mining ineffective. We have applied a recently developed pipeline for HRGP/AGP mining, ragp, which is based on machine learning prediction of proline hydroxylation, to identify HRGP sequences in centaury transcriptome and to classify them into motif and amino acid bias (MAAB) classes. AGP sequences with low AG glycomotif representation were also identified. Six members of each of the three AGP subclasses, fasciclin-like AGPs, receptor kinase-like AGPs and AG peptides, were selected for phylogenetic and expression analyses. The expression of these 18 genes was recorded over 48 h following leaf mechanical wounding, as well as in 16 tissue samples representing plants from nature, plants cultivated in vitro, and developmental stages during shoot organogenesis and somatic embryogenesis. None of the selected genes were upregulated during both wounding recovery and regeneration. Possible functions of AGPs with the most interesting expression profiles are discussed.

## 1. Introduction

Hydroxyproline-rich glycoproteins (HRGPs) are a super family of plant cell surface O-linked glycoproteins. They are embedded into the cellulose/hemicellulose and pectic polysaccharide networks of the cell wall and are involved in the cell dynamics through diverse functions in growth and development, environmental sensing, signaling and defense [1,2,3]. Based on the patterns of proline hydroxylation and subsequent glycosylation, HRGPs are traditionally classified into three families: the highly glycosylated arabinogalactan proteins (AGPs), the moderately glycosylated extensins (EXTs) and the lightly glycosylated proline-rich proteins (PRPs). The distinction among these groups is often blurred, since many sequences have shared characteristics. To acknowledge this, the motif and amino acid bias (MAAB) classification system was developed which classifies HRGPs into 23 descriptive subclasses with an additional 24th class containing sequences with high amino acid bias but low motif coverage [4]. Among the three classical HRGP families, AGPs have attracted considerable attention due to their structural diversity and roles in many physiological processes [5].

The prototypical AGP molecule consists of a protein backbone rich in amino acids P, A, S and T, arranged in dipeptide motifs PA, PS, PT, AP, SP and TP (likely also VP, PV, GP and PG) so-called AG motifs or AG glycomodules. Prolines in AG motifs can be posttranslationally modified by prolyl-hydroxylases into hydroxyprolines (O) which serve as attachment sites for branched type II arabinogalactan (AG) polysaccharides [5]. The attached oligosaccharides can account for more than 90% of the total AGP mass. In addition, many AGPs are attached to the cell membrane via a glycosylphosphatidylinositol (GPI) anchor [5]. Since proline disrupts secondary structures, AGPs lack a stable hydrophobic core and are intrinsically disordered proteins (IDPs); as a consequence, the evolutionary constraints imposed on AGP sequences are lessened, which in turn limits the success of homology-based identification. Traditionally, AGPs have been identified via high frequencies of PAST amino acids, machine learning prediction of N-terminal secretory signal sequences (N-sp) and C-terminal GPI attachment signals [6]. Some AGP sequences contain basic amino acid (H and/or K) spans [7,8]. Short AGP sequences, arabinogalactan peptides (AGp), with a length shorter than 90 amino acids (including N-sp and GPI signal sequences), have also been identified [6]. A subset of these contains a conserved region (PF06376, formerly domain of unknown function—DUF1070) encompassing the short PAST-rich span and a conserved GPI signal [9,10]. Additionally, certain protein families with domains such as fasciclin (protein family database (Pfam) accession: PF02469), nonspecific lipid transfer protein (PF00234, PF14368), plastocyanin-like (PF02298), pollen protein Ole e 1-like (PF01190, also referred to as proline-rich arabinogalactan protein and conserved cysteines—PAC) and leucine-rich repeats (LRR, Pfam: CL0022) are often associated with AGP features. Thus, a magnitude of so-called chimeric AGPs, representing a combination of a conserved domain and AGP spans, have been described [6,11]. More recent bioinformatics sequence analysis, enabling identification of short AGP-like sequence spans, has revealed characteristic AGP features in even more protein families, such as the protein tyrosine and serine/threonine kinase (PF07714), X8 (PF07983), glycoside hydrolase family 17 (PF00332), salt stress response/antifungal (PF01657) and others [12,13].

Given their structural heterogeneity, it is not surprising that AGPs perform diverse physiological functions [14]. AGPs are implicated in cell proliferation, embryogenesis, plant growth, reproduction, secondary wall deposition, xylem differentiation, programmed cell death, hormone responses and abscission [5,14,15,16,17]. AGPs have roles in response to different types of stress: osmoregulation [18], chilling tolerance [19], wounding [20,21,22] and plant-microbe interactions [23]. In addition, a large number of sequences having AG motifs on the extracellular side and an intracellular receptor kinase domain (PF07714—protein tyrosine and serine/threonine kinase) have been identified using a bioinformatics approach in the 62 studied plant proteomes [13], suggesting that AGP regions in these sequences might be implicated in signaling in a very direct manner.

*Centaurium erythraea* Rafn (Gentianaceae), European, common or small centaury, a medicinal plant rich in secoiridoid glucosides and xanthones, is traditionally used in treating gastrointestinal disorders, anemia and other conditions. *C. erythraea* extracts also possess hepatoprotective, diuretic, anti-inflammatory, antioxidative, antibacterial and antifungal properties [24]. *C. erythraea* is characterized with extraordinary developmental plasticity and manageability in vitro and vigorous, often spontaneous, regeneration via different morphogenic paths [25,26]. Studies based on interaction of AGPs with β-D-glucosyl Yariv reagent (βGlcY), which selectively binds AGPs causing precipitation [10,27,28], or with antibodies against AGPs’ glycan moieties [29], have identified AGPs as one of the key players involved in somatic embryogenesis (SE) and shoot organogenesis (SO) in centaury. One of the inevitable consequences of plant tissue in vitro manipulations is mechanical wounding. Wound signaling initiates both defense responses and healing responses regenerative cell reprogramming, which includes dedifferentiation, cell cycle reactivation and vascular regeneration [30]. Thus, under in vitro conditions, wounding may stimulate induction of SE [31,32] and SO [33]. However, the possible role of AGPs in wounding responses has not been investigated in centaury so far.

We have recognized the value of *C. erythraea* as a potential novel model organism for developmental biology studies [25], but the lack of genetic sequence information has until recently restrained research at the molecular level [34]. The transcriptomic resources [34], combined with the development of the ragp bioinformatics pipeline [13] have enabled an in depth look at the *C. erythraea* AGP family presented here. One of the goals of the current study was to identify centaury AGPs involved in SE and SO, as well as AGPs involved in wounding response, since wounding may stimulate SE and SO, as mentioned above. To achieve this, we have first identified AGPs present in the transcriptome and then focused on 18 AGP gene representatives—six from each of the three subclasses: fasciclin-like AGPs (FLA), receptor kinase-like AGPs (as a novel AGP type these are termed here as kinase-like AGPs or KLAs) and AG peptides (AGp). Certain FLAs were previously indicated in centaury SE [10] and are also known to be involved in zygotic embryogenesis in *A. thaliana* and other species [17] and for this reason were selected for further investigation. An AGp with the recently described PF06376 domain was also found to be implicated in centaury morphogenesis in vitro [10]; therefore, we have analyzed AGps with and without this conserved domain. Finally, recently described KLAs are interesting as an underexplored group of possible signaling molecules. Temporal expression patterns were monitored for the selected 18 genes after mechanical wounding of leaf explants, along with their expression in tissue samples representing the stages of SE and SO, with a number of other samples (from in vitro culture and from plants from nature, see [34]) which were analyzed for comparison. Structure, phylogenetic relations and possible functions of the selected AGPs are discussed.

## 2. Results

### 2.1. Motif and Amino Acid Bias (MAAB) Classification of Centaury HRGP Sequences

**Figure 1:**
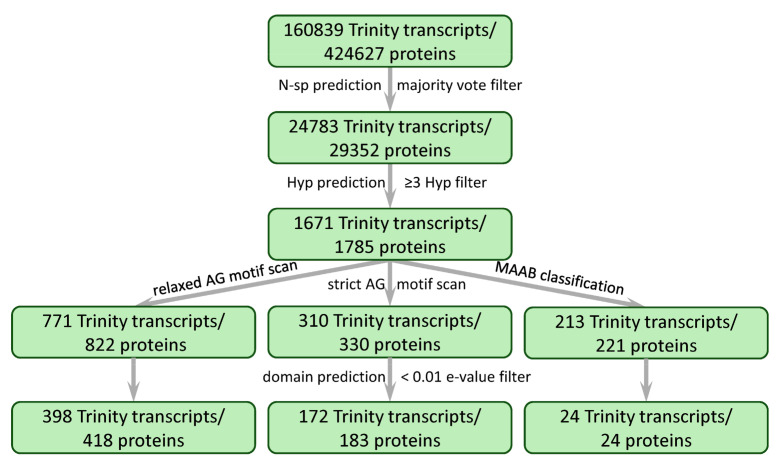
The number of *C. erythraea* Trinity transcripts and predicted protein sequences passing through different filters of the ragp pipeline.

**Figure 2:**
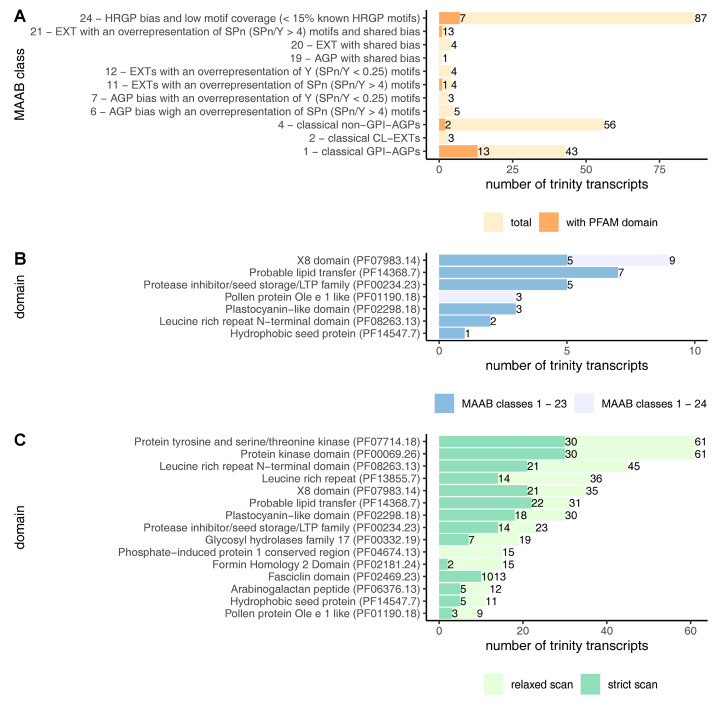
Distribution of identified HRGP sequences in *C. erythraea* transcriptome. (**A**) Distribution of HRGP MAAB classes. (**B**) Distribution of Pfam domains in sequences identified as one of the MAAB classes. (**C**) Distribution of Pfam domains (top 15 by frequency) in sequences containing clusters of AG motifs, using a relaxed scan (three AG motifs no more than 10 amino acids apart) and a strict scan (four AG motifs no more than four amino acids apart). Unique domains per sequence were counted. Only AG motifs with prolines likely to be hydroxylated were considered. AG motifs linked to three or more continuous prolines/hydroxyprolines were omitted from the scan (example AOOO). Domains were predicted with hmmscan 3.3.2 and using Pfam-A33 database using a cutoff of 0.01 for independent e-value.

As a starting point for the identification of *C. erythraea* HRGP sequences, the *de novo* Trinity-assembled transcriptome was used [34]. Trinity assembled 160839 transcript sequences and grouped the closely related transcripts (ideally alternatively spliced isoforms, but often transcripts belonging to paralogues genes and chimeric transcripts) into 105726 genes. The referent transcriptome was analyzed using ragp—a recently developed bioinformatics pipeline that combines machine learning-based prediction of hydroxyproline sites with other HRGP mining tools [13]. The first instance for the filtration of HRGPs, as cell wall proteins, is the presence of N-sp. The ragp pipeline incorporates N-sp prediction based on majority vote among Phobius 1.01, SignalP 4.1 and TargetP 1.1. Protein products from 24783 Trinity transcripts were selected this way (Figure 1). Prediction of proline hydroxylation probability in these protein sequences identified 1785 protein products from 1671 Trinity transcripts (1063 Trinity genes) as likely to contain at least three hydroxyprolines. The modified MAAB classification pipeline [4] incorporated in ragp identified 221 protein sequences (from 213 Trinity transcripts, Supplementary Table S1) as belonging to one of the 1–24 MAAB classes (Figure 1 and Figure 2A). The majority of these sequences (87 Trinity transcripts) belong to MAAB class 24—sequences with HRGP bias and low representation of known HRGP motifs (less than 15% of the sequence is covered with known HRGP motifs). From the remaining 126 Trinity transcripts belonging to MAAB classes 1–23, the majority of sequences were classified as classical AGPs (99 Trinity transcripts) with predicted GPI signal peptide (class 1, 43 Trinity transcripts) and classical AGPs without predicted GPI signal (class 4, 56 Trinity transcripts), while the rest of the MAAB classes were either absent or represented with few sequences. The most frequent domains identified in these sequences were probable lipid transfer (Pfam: PF14368.7), protease inhibitor/seed storage/LTP family (PF00234.23) and X8 (PF07983.14), which was particularly frequent in MAAB class 24 (Figure 2B). The majority of the mentioned domains were identified in classical AGPs with predicted GPI signal peptide (class 1, Figure 2A). It should be noted that domain pollen protein Ole e 1-like (PF01190.18) was identified only in sequences classified as MAAB class 24 (Figure 2B).

### 2.2. Identification of AGP Sequences with Low AG Glycomotif Representation

To explore the diversity of the sequences containing AG motifs, regardless of the compositional bias, two types of ragp AG motif sequence scans were performed: a relaxed scan, where sequences with at least three AG motifs no more than ten amino acids apart were identified, and a more stringent scan, where sequences with at least four AG motifs no more than four amino acids apart were identified (Figure 2C, Supplementary Table S2). It should be noted that only AG motifs with prolines likely to be hydroxylated, as predicted by ragp, were considered, and that motifs with three or more continuous prolines/hydroxyprolines (motifs such as AOOO or OOOOS) were not considered as AG motifs. Approximately 40% of the sequences (310 of 771 Trinity transcripts or 330 of 822 Protein sequences) identified with the relaxed scan were also present after the stricter AG motif scan (Figure 1). The most frequently identified domains in both groups of sequences (relaxed and strict scan) were the kinase domains (PF07714.18 and PF00069.26), followed by LRR (PF08263.13 and PF13855.7). Interestingly, none of the sequences identified with the relaxed scan that have the phosphate-induced protein 1 conserved region (PF04674.13), and very few with the formin homology 2 domain (PF02181.24) passed the strict scan, indicating that these sequences probably are not chimeric AGPs, or that they contain short AG motif spans made from three dipeptides.

### 2.3. Structural Features of AGP Sequences Selected for Expression Analysis

**Figure 3:**
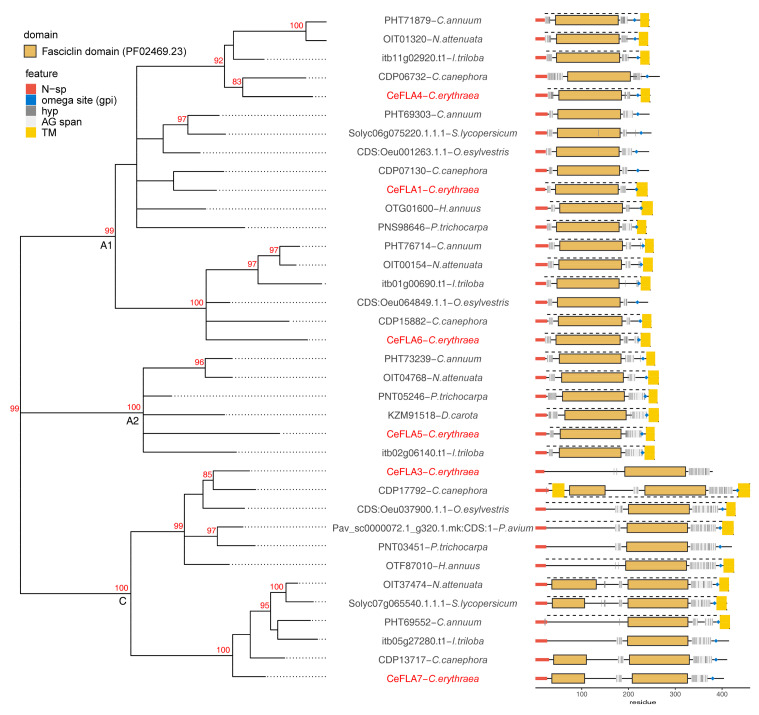
Phylogenetic relationship of six *C. erythraea* FLA protein sequences with homologues from other plant species. The phylogenetic tree represents an unrooted maximum likelihood tree constructed using the WAG amino acid model. Cluster stability was evaluated using 100 replicates of nonparametric bootstrap. Clusters with ≥80/100 bootstrap support are indicated with a red number. Clusters with ≤50/100 bootstrap support were collapsed into multichotomies. Protein schematic diagrams were constructed using ragp R package: N-sp as predicted by Signalp4.1 are represented with red segments on the N-terminus; GPI addition sites (omega sites) as predicted using NetGPI1.1 are represented with blue diamonds; domains as predicted with hmmscan 3.3.2 and using Pfam-A33 database are represented according to the color legend; transmembrane domains (TM) as predicted using Phobius1.01 are represented using yellow rectangles. Proteins with predicted TM extracellular regions are indicated with dashed lines above the sequence diagrams; hydroxyprolines, as predicted with ragp 0.32 are indicated with bar-code-like vertical black lines, while AG motif spans (at least three AG motifs, no more than 10 amino acids apart) are indicated with light grey rectangles. Clades are labeled according to [35].

**Figure 4:**
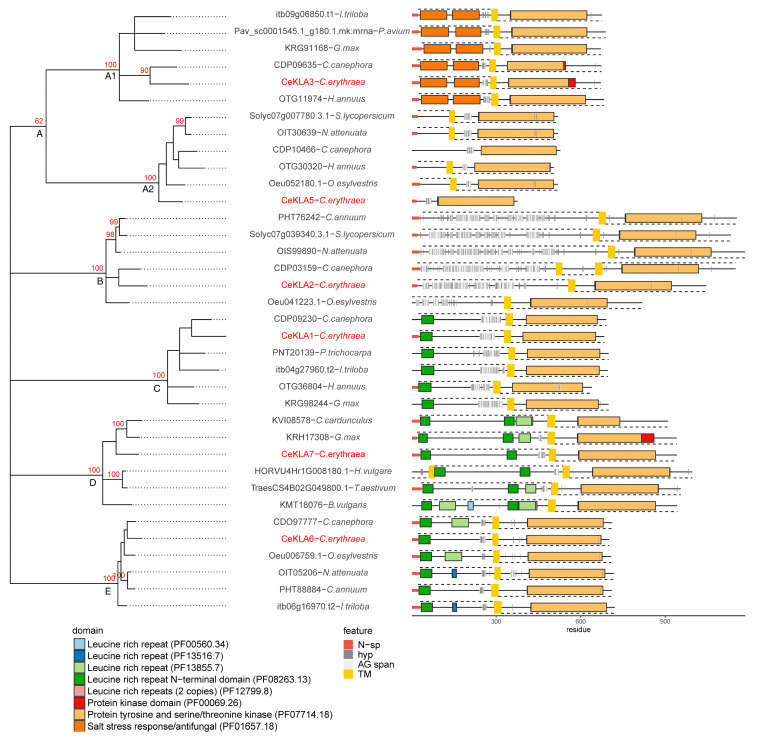
Phylogenetic relationship of six *C. erythraea* KLA protein sequences with homologues from other plant species. The phylogenetic tree represents an unrooted maximum likelihood tree constructed using the JTT amino acid model. Cluster stability was evaluated using 100 replicates of nonparametric bootstrap. Clusters with ≥80/100 bootstrap support are indicated with a red number. Clusters with ≤50/100 bootstrap support were collapsed into multichotomies. Intermediate (50–80) bootstrap support is indicated on major clusters. Protein schematic diagrams were constructed using ragp R package: N-sp as predicted by Signalp4.1 are represented with red segments on the N-terminus; domains as predicted with hmmscan 3.3.2 and using Pfam-A33 database are represented according to the color legend; transmembrane domains (TM) as predicted using Phobius1.01 are represented using yellow rectangles. Proteins with predicted TM extracellular regions are indicated with dashed lines above the protein diagram, while the intracellular regions are indicated with dashed lines below the protein diagrams; hydroxyprolines as predicted with ragp 0.32 are indicated with bar-code-like vertical black lines, while AG motif spans (at least three AG motifs, no more than 10 amino acids apart) are indicated with light grey rectangles.

**Figure 5:**
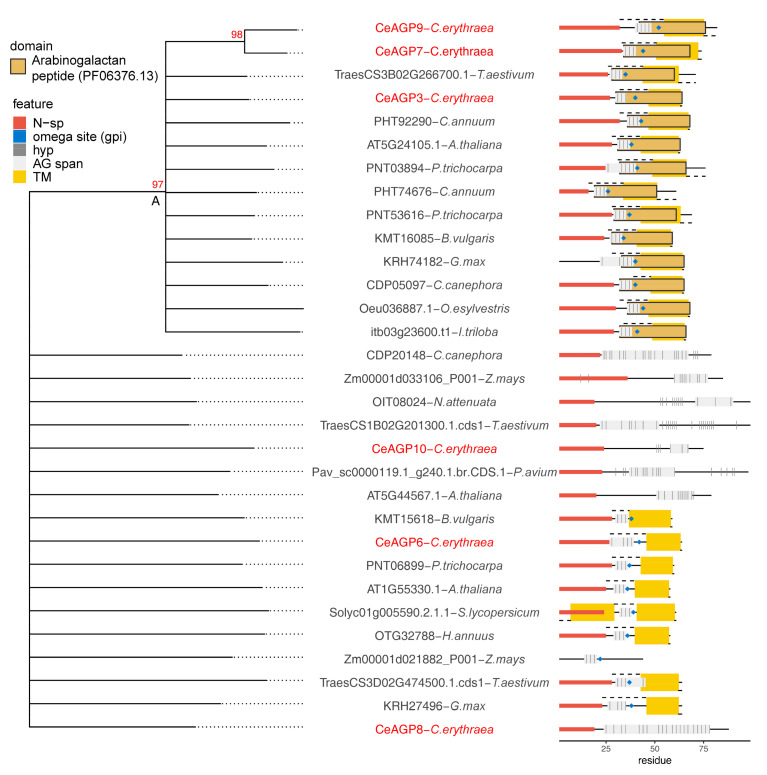
Phylogenetic relationship of six *C. erythraea* AGp protein sequences with homologues from other plant species. The phylogenetic tree represents an unrooted neighbor joining tree constructed based on an alignment-free distance matrix (3-mer count as implemented in kmer R package). Cluster stability was evaluated using 100 replicates of nonparametric bootstrap. Clusters with ≥80/100 bootstrap support are indicated with a red number. Clusters with ≤50/100 bootstrap support were collapsed into multichotomies. Protein schematic diagram was constructed using ragp R package: N-sp as predicted by Signalp4.1 are represented with red segments on the N-terminus; GPI addition sites (omega sites) as predicted using NetGPI1.1 are represented with blue diamonds; domains as predicted with hmmscan 3.3.2 and using Pfam-A33 database are represented according to the color legend; trans-membrane domains (TM) as predicted using Phobius1.01 are represented using yellow rectangles; proteins with predicted TM extracellular regions are indicated with dashed lines above the sequence diagrams, while intracellular regions are indicated with dashed lines below the sequence diagrams; hydroxyprolines as predicted with ragp 0.32 are indicated with bar-code-like vertical black lines, while AG motif spans (at least three AG motifs, no more than 10 amino acids apart) are indicated with light grey rectangles.

From the identified sequences with AG motifs, we have chosen 18 transcripts with well-defined AG motif clusters, for which specific primers could be constructed to quantify just the corresponding Trinity transcript, which were examined in more depth (Supplementary Table S3). Six representatives from each of the KLA, FLA and AGp classes of *C. eryhtraea* sequences were chosen, of which three featured the recently described arabinogalactan peptide domain—PF06376 [9]. The phylogenetic relationship of these sequences to similar sequences from eighteen plant species was studied (Figure 3, Figure 4 and Figure 5). In order to provide context to the generated phylogenetic trees, a protein structure diagram was generated for sequences from each tree. The FLA tree consists of three major clusters, A1, A2 and C (Figure 3). Sequences from A1 and A2 belong to the FLA group A, while sequences from C belong to FLA group C based on similarity to corresponding Arabidopsis FLA [35] of both full-length sequences and isolated FAS domains. The cluster A1, with *C. eryhtraea* CeFLA4, CeFLA1 and CeFLA6 sequences, is comprised of sequences with one fasciclin (FAS) domain and two AGP regions, one before and one after the FAS domain (Figure 3). The cluster A2 consists of sequences structurally similar to sequences in A1, while cluster C consists of longer FLA sequences comprised of either one FAS domain closer to the C-terminus, or two FAS domains (Figure 3). Sequences from C with two FAS domains (like CeFLA7) contain two AG motif spans: one shorter between the two FAS domains and one longer C-proximal AG motif span, while C group sequences with one FAS domain (like CeFLA3) contain a shorter AG motif span before the FAS domain and a longer span after it. Four of the analyzed *C. eryhtraea* FLAs (CeFLA4, CeFLA1, CeFLA3 and CeFLA6) are phylogenetically closest to *Coffea canephora* FLAs, which is not surprising since out of the 18 considered plant species, *C. canephora* is taxonomically closest to *C. eryhtraea*, both belonging to the order Gentianales. All considered FLAs, except CeFLA3, contain a predicted omega site (GPI attachment site), which is likely because CeFLA3 is a partial sequence without a complete C-terminus.

The KLA phylogenetic tree (Figure 4) is comprised of five clusters A–E. The majority of KLA sequences feature a TM region approximately in the middle of the sequence, followed by a C-terminal intracellular kinase region. The cluster A with intermediate bootstrap support (62/100) consists of two stable clusters, A1 and A2. A1 is comprised of sequences containing two salt stress response/antifungal domains on the extracellular N-terminal side, after which there is a short hydroxyproline span just prior the TM. In three sequences, CeKLA3-*C. erythraea*, CDP09635-*C. canepohora* and OTG11974-*H. annuus*, the predicted hydroxyprolines are in the AG motif context. A2 subcluster contains sequences lacking domains other than kinase, with relatively odd architecture: two sequences lack TM (CeKLA5 and CDP10466−*C. canephora*), while four sequences have a predicted TM, but the predicted AG motif spans and hydroxyprolines are in the intracellular regions; this finding should either be discarded or considered with great caution, since the ragp prediction model was trained only on extracellular sequence regions. Cluster B is comprised of proline-rich receptor kinase sequences with a long HRGP-like extracellular region containing mixed AG and extensin motifs, as represented by CeKLA2. Clusters C, D and E consists of KLA with LRR in the extracellular region (like *C. eryhtraea* CeKLA1, CeKLA6 and CeKLA7). Cluster C (with CeKLA1) is comprised of sequences with one N-terminal LRR region and a long AG motif span preceding the TM region. Cluster D (with CeKLA7) is comprised of long KLA sequences, with several LRR regions and vague AGP characteristics (some sequences have short AG spans, while others such as KVI08578—*C. cardunculus* and KMT18076—*B. vulgaris* lack AG motifs altogether). Cluster E (with CeKLA6) is comprised of sequences with one or two LRR regions and short pre-TM AG motif spans, while one sequence (Oeu006759.1−*O. esylvestris*) has only an extensin region. Similarly to the FLA phylogenetic tree (Figure 3), four of the six studied *C. erythraea* KLA protein sequences, CeKLA6, CeKLA1, CeKLA3 and CeKLA2, cluster tightly with KLA sequences from *C. canephora*, as a consequence of the taxonomical proximity of these two species.

The phylogenetic tree for shorter AGP sequence or AG peptides (AGp) (Figure 5) was constructed using an alignment-free approach, based on 3-mer amino acid occurrence, due to the high divergence of the sequences. This divergence is also illustrated by the fact that only one cluster, A, (Figure 5) comprised of sequences with the arabinogalactan peptide (PF06376.13) domain was stable (high bootstrap support), while the remaining sequences were collapsed into a multichotomy (less than 50/100 bootstrap support). Cluster A is comprised of sequences with a predicted GPI attachment site and a short AG motif span just between the N-sp and omega site. The remaining sequences consist of either AGp with short AG motif spans and a predicted GPI attachment site (like CeAGP6) and AGp with longer AG motif spans which are in some cases combined with extensin motifs (like CeAGP10 and CeAGP8), for which the presence of the GPI attachment site was not predicted. However, both CeAGP8 and CeAGP10 are partial lacking a C-terminus, so it is possible they are not AG peptides, but rather longer AGPs.

### 2.4. Wounding Response of AGP Genes

**Figure 6:**
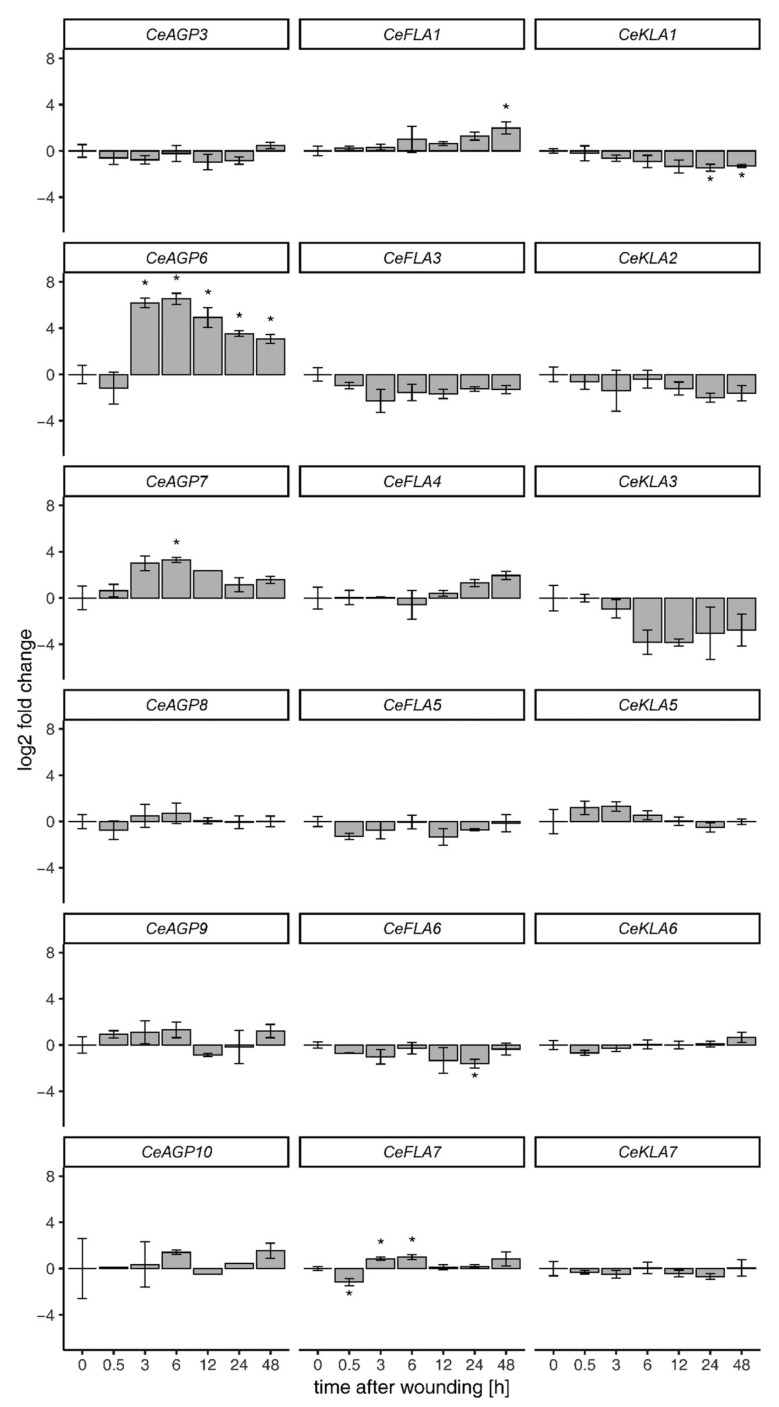
Temporal gene expression profiles for the 18 studied AGp, FLA and KLA genes obtained after cutting of leaf explants. Mean and standard deviation is shown for three biological replicates. Immediately frozen tissue after wounding (0 min) was used as control sample. Asterisks indicate significant difference obtained via Student’s *t*-test between a specific time point and the appropriate control sample. Multiple comparison correction was performed jointly for all performed comparisons using the Benjamini and Holcberg method.

In order to explore if any of the analyzed genes are involved in *C. erythraea* wounding response, gene expression was recorded over a period of 48 h after cutting the leaf explants (Figure 6). Six of the studied *C. erythraea* genes, *CeAGP6*, *CeAGP7*, *CeFLA1*, *CeFLA6*, *CeFLA7* and *CeKLA1* had statistically significant expression changes as compared to the tissue samples frozen immediately upon cutting (0 min, control sample, Figure 6). However, most of the studied genes had only mild changes in expression, and only *CeAGP6* and *CeAGP7* stood out as highly and immediately induced genes. *CeAGP6* was upregulated almost 100-fold (6.5 log2 fold change 6 h after wounding), while *CeAGP7* was upregulated almost 10-fold (3.3 log2 fold change also 6 h after wounding). Both genes shared similar expression profiles, with modest changes in expression 30 min after wounding, a sharp increase 3 h after wounding and both peaking 6 h after wounding, after which the expression declined steadily. From the rest of the studied genes, the downregulation of *CeKLA3* was notable, although its changes in expression were not statistically significant due to high variance in expression of biological replicates and the correction for the number of statistical comparisons. Based on the trends of expression, perhaps notable are also *CeFLA1* and *CeKLA1*, which showed a stable, yet modest change in expression after wounding, with opposite trends, both peaking near the end of the studied period. *CeFLA1* steadily increased in expression, reaching a peak 48 h after wounding at 2 log2 fold change, while *CeKLA1* steadily declined in expression, reaching a peak of –1.5 log2 fold change, 48 h after wounding.

### 2.5. Expression of AGP Genes in Centaury Organs and in Different Developmental Stages during Organogenesis and Somatic Embryogenesis in Vitro

We have also quantified the expression of the 18 AGP genes in sixteen samples (Table 1) encompassing organs from in vitro grown plants (whole seedlings, leaves and roots from plants at the rosette stage), in vitro grown root cultures, organs from flowering plants collected from nature (leaves, roots, stems, immature and mature flowers), as well as tissue samples representing the processes of organogenesis (organogenic calli and adventitious buds of different origin) and SE (embryogenic calli and globular and cotyledonary somatic embryos). Both morphogenic paths occur in the presence of 0.2 mgl^−1^ 2,4-dichlorophenoxyacetic acid (2,4-D) and 0.5 mgl^−1^ N-(2-chloro-4pyridyl)-N′-phenylurea (CPPU) in light, but organogenesis may also occur spontaneously on hormone-free medium, while SE, unlike organogenesis, may also proceed in the darkness [25,34].

**Figure 7:**
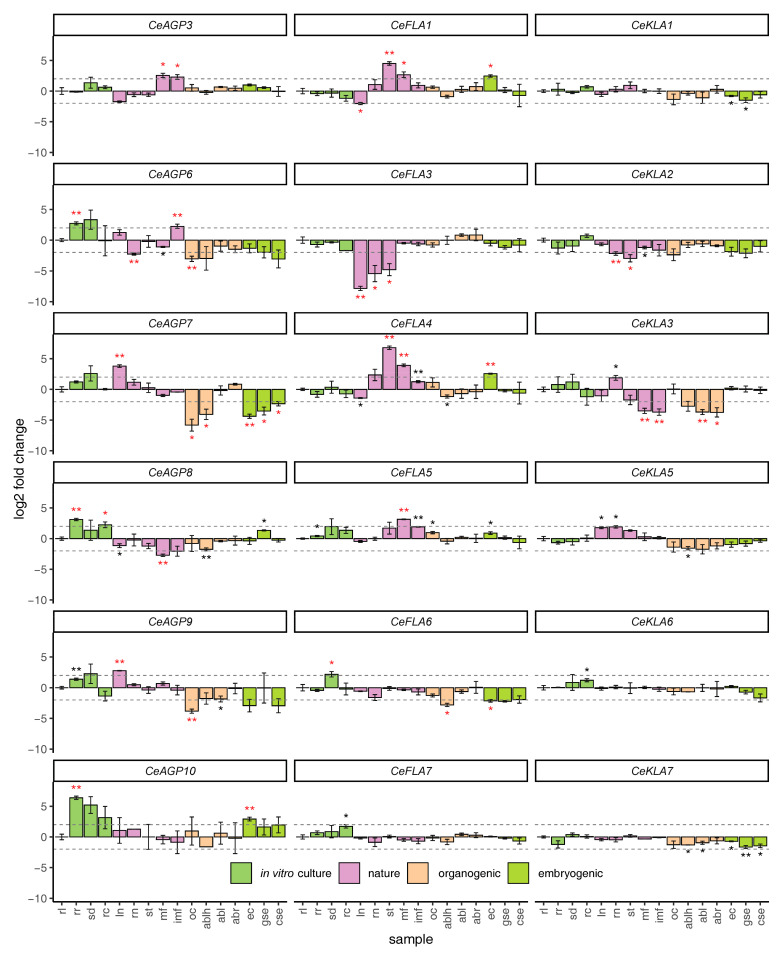
Expression profiles for the 18 studied *AGp*, *FLA* and *KLA* genes in 16 different developmental stages, tissues and plant organs. Sample abbreviations are provided in Table 1. Mean and standard deviation is shown for three biological replicates. The samples correspond to the experimental system explained in more depth in [34]. The horizontal dashed lines represent a log2 fold change of 2 and −2 compared to the rosette leaves (rl) grown in vitro. Statistical comparison of the means was performed between each sample and the rosette leaves (rl) grown in vitro (control sample) using Welch’s *t*-test. Multiple comparison correction was performed jointly for all performed comparisons using the Benjamini and Holcberg method. One asterisk (*) indicates that the mean expression in the sample is significantly different compared to the mean control expression (adjusted *p*-value < 0.05), while two (**) correspond to highly significant difference in expression (*p*-values < 0.01). Red asterisks indicate that the comparison of the means is significant and that the difference in the means is higher than 2 log2 fold.

The expression of all studied genes was significantly different in at least some samples compared to the rosette leaf (rl) used as the control sample (Figure 7). For some comparisons, the obtained statistically significant differences were impacted more by the low variance of expression in the samples, especially the control sample for certain genes, rather than the overall differences in average expression (most of the *CeKLAs*, *CeFLA6*, *CeFLA7* and *CeAGP3*, Figure 7); therefore, we restrict our analysis to the statistically significant differences which are at least 2 log2 fold different compared to the control (Figure 7). From the 18 analyzed genes, 13 had more than 2 log2 fold changed expression in any of the analyzed samples as compared to the rosette leaf (Figure 7). These 13 genes included all of the analyzed AGp genes, all FLA genes except *CeFLA7* and two KLA genes, *CeKLA2* and *CeKLA3*. Interestingly, both of the genes that were induced upon wounding—*CeAGP6* and *CeAGP7* were downregulated in the majority of embryogenic and organogenic samples. This was more pronounced for *CeAGP7*, where five of the seven embryogenic and organogenic samples had significantly downregulated expression: organogenic calli (oc), leaf-derived adventitious buds developed on 2,4-D/CPPU medium (ablh, embryogenic calli (ec), globular and cotyledonary somatic embryos (gse and cse) developed on the same medium (see Table 1). *CeAGP7* had significantly upregulated expression in leaves collected from plants grown in nature (ln). *CeAGP6* was significantly downregulated in organogenic calli (oc) and roots from flowering plants from nature (rn), while it was upregulated in rosette roots (rr) and immature flowers from plants grown in nature (imf). *CeAGP9* expression was significantly decreased in oc and upregulated in leaves from plants grown in nature (ln). CeAGP10 was highly expressed in rr. Apart from these AGp genes, the biggest changes in expression as compared to controls were observed for *CeFLA3*, which was generally poorly expressed in vegetative organs from plants from nature—leaves (ln), roots (rn) and stems (st). In contrast to *CeFLA3*, the genes *CeFLA1*, *CeFLA4* and to lesser extent *CeFLA5* were generally more expressed in plants grown in nature than in culture in vitro. *CeKLA3* had the lowest expression in adventitious buds regardless of their origin, as well as in mature and immature flowers (mf and imf) from plants grown in nature. Finally, several genes such are *CeFLA7, CeKLA1, CeKLA6* and *CeKLA7* showed pretty much constitutive expression (less than 2 log2 fold change compared to rl in all samples) across the tested samples (Figure 7).

**Figure 8:**
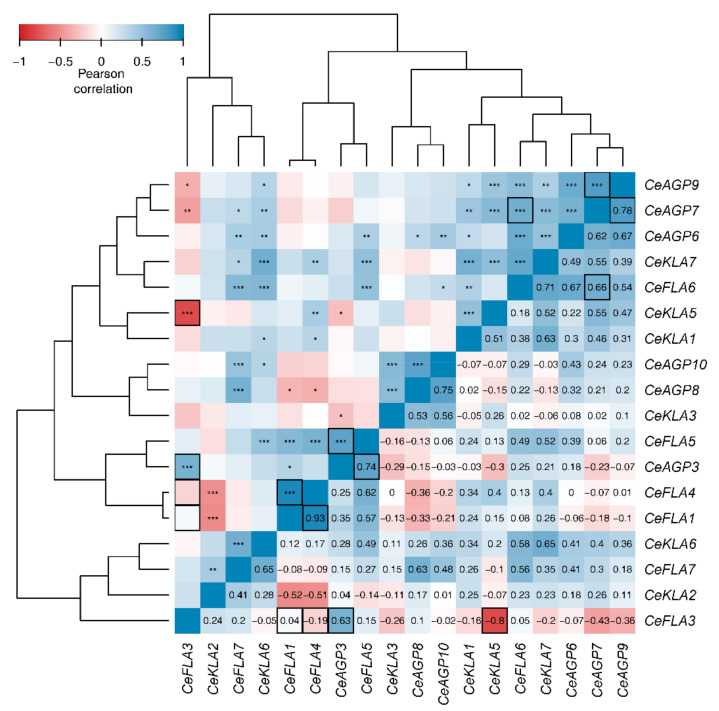
Pearson correlation heatmap of relative gene expression. Pairwise correlation coefficients are given in the lower diagonal triangle, while statistical significance of the pairwise association is shown in the upper triangle: * for *p*-value < 0.05, ** for *p*-value < 0.01 and *** for *p*-value < 0.001. Rows and columns of the heatmap are arranged according to hierarchical cluster analyses performed on a matrix of correlation distances (1–cor_Pear_). Cluster agglomeration was performed using complete linkage and the dendrograms are shown on top and left of the heatmap. Emphasized cells (enclosed with rectangles) correspond to the top 5% pairs based on Jackknife bias corrected mutual information.

Pearson correlation was used to estimate the linear relations among the genes based on their expression in 16 plant tissue samples (Figure 8). An almost linear dependence was found for *CeFLA1* and *CeFLA4* (0.93 cor_Pear_), while a negative correlation was found for *CeKLA5* and *CeFLA3* (−0.8 cor_Pear_). High positive correlations were found among genes *CeAGP9*, *CeAGP7*, *CeAGP6*, *CeKLA7* and *CeFLA6* (Figure 8), all of which are to some extent downregulated in samples representing SE and SO and upregulated in seedlings (Figure 7). To further explore the relationship among genes based on their expression profiles, we quantified how much information the expression pattern of each gene holds about all others in a pairwise manner, using bias corrected mutual information (BCMI). In the top 5% percent of gene pairs, in regards to BCMI, the majority were the pairs with high absolute Pearson correlation: *CeAGP7* and *CeAGP9*, *CeAGP7* and *CeFLA6*, *CeAGP3* and *CeFLA5*, *CeFLA3* and *CeKLA5* and *CeFLA3* and *CeAGP3*. In addition, the pairs *CeFLA3* with *CeFLA1* and *CeFLA4* had high BCMI (top 5%) and low Pearson correlation, indicating a non-linear relationship exists between these gene expression patterns.

## 3. Discussion

### 3.1. Size and Diversity of C. erythraea HRGP Superfamily

Several approaches to bioinformatics identification of potential HRGP, and specifically AGP sequences, have been developed in recent years in an attempt to overcome limitations of classical approaches to HRGP/AGP mining. The MAAB classification system was developed to acknowledge the existence of a continuum of HRGP sequences with shared characteristics of extensins, AGPs and proline-rich proteins [4]. On the other hand, the ragp pipeline was created to detect AGP sequences with underrepresentation of AG motifs, by combining machine learning-based hydroxyproline prediction with hydroxyproline-aware AG motif scans [13]. Therefore, both approaches were employed to investigate the diversity of centaury HRGPs and especially AGP sequences. By using the modified MAAB pipeline, as incorporated in ragp, on predicted protein sequences from the *de novo* assembled *C. erythraea* transcriptome [34] (https://zenodo.org/record/3591805, accessed on 12 August 2021), 126 Trinity transcripts (134 protein sequences) were classified into MAAB HRGP classes 1–23, while the remaining 87 transcripts (corresponding to the same number of proteins) were classified as MAAB class 24 (Figure 1 and Figure 2A and Supplementary Table S1). Among the 62 Phytozome V12 proteomes analyzed using an equivalent approach [13], only five plant or algae species (*Chlamydomonas reinhardtii, Hordeum vulgare*, *Physcomitrella patens*, *Triticum aestivum* and *Zea mays*) were shown to contain more than 100 protein sequences classified as MAAB 1-23. A combination of biological and technical causes can conceivably explain the high number of MAAB 1-23 classified sequences as compared to Phytozome proteomes. Namely, BUSCO assessment [36] of the *C. erythraea* transcriptome indicated a very high (95%) completeness, but also a high percentage of duplicated complete BUSCOs (54%), likely because the sequenced centaury was a tetraploid [34]. The heterozygous alleles that failed to collapse during *de novo* assembly, probably causing the observed duplications, might as well be responsible for the high number of MAAB 1-23 classified sequences. Separating out alleles and paralogs is a challenge during *de novo* assembly, especially for polyploid organisms, and Trinity, like other short-read *de novo* assemblers, can generate chimeras between them [37,38], leading to additional inflation of the redundancy. Even though there are methods for redundancy reduction [37,39,40], we have not applied them, to avoid losing real biological entities (sequences). Finally, the comparison of Phytozome V12 proteomes, which are generated based on reference haploid genomes, with a proteome created from a *de novo* assembled transcriptome can produce only rough estimates.

The majority of *C. erythraea* sequences within MAAB classes are classical AGPs (classes 1 and 4), while other classes, apart from class 24, are either underrepresented or absent (Figure 2A and Supplementary Table S1). This is in concordance with the finding that in eudicot transcriptomes class 24 has the highest average number of sequences, followed by classes 4 and 1, while other MAAB classes are underrepresented [41]. However, the number of MAAB classified sequences in each group is much higher in our work compared to [41], probably because we did not exclude sequences with predicted domains or short (<90 aa) protein sequences. Another potential reason is sequence redundancy, as pointed above. The fact that the majority of MAAB 1–23 classified sequences belong to classical AGPs (MAAB classes 1 and 4) may not necessarily imply that centaury lacks diversity of other HRGP classes. Johnson et al. [41] have shown that when analyzing *de novo* transcriptomic data for MAAB classification, it is critical to employ a multiple k-mer assembly. When using a 25-mer approach, which is the default for many De Bruijn graph transcriptome assemblers, including Trinity, which we have used [34], they observed an underrepresentation of the highly repetitive HRGP sequences such as CL-EXTs and PRPs. Since we were interested in recovering AGP sequences, particularly those with few AG motifs, k-mer size used for *de novo* assembly should not have a high impact on the recovered sequences, as evidenced by the high number of identified sequences. Additionally, the distribution of the MAAB classes is partly obscured because a portion of protein sequences lacks a complete C-terminus; thus, the GPI prediction, which is required for resolving MAAB classes, can be misleading.

Considering that MAAB classification is based on the presence of glycosylation motifs in HRGPs and on the high percentage of amino acids that constitute these motifs (amino acid bias), such as a threshold of over 45% of P, A, S and T in the case of AGPs [4], it is not surprising that MAAB-recognized sequences have few, usually shorter Pfam domains or none at all (Figure 2A). This is simply because the presence of larger domains, such as fasciclin (PF02469), which is commonly associated with AGPs [19,42], would diminish the amino acid bias of these sequences. Thus, only a few MAAB classes, most notably classical GPI-AGPs (class 1), were found to have any Pfam domains (Figure 2A). Among these, some are prominent and well-known AGP associates, such are nonspecific/probable lipid transfer protein (PF00234 and PF14368) and plastocyanin-like domain (PF02298), while others, like X8 domain, have only recently been associated with AGPs [12,13,43] (Figure 2B).

Apart from finding HRGP representatives via MAAB classification, we were particularly interested in identifying sequences which potentially contain short AGP-like sequence spans, a task that ragp R package was created for. A number of sequences were identified with both the relaxed and strict scans (822 and 330 protein sequences, respectively, Figure 1), many of them associated with different Pfam domains (Figure 2C, Supplementary Table S2). In addition to the relaxed AG motif scan [13], in this work we have adopted an additional stricter AG motif scan, in order to identify potentially false positive sequences. Approximately 40% of all protein sequences filtered with the relaxed scan were also found with the strict scan. Since no sequences containing PF04674.13 (phosphate-induced protein 1 conserved region) passed the stricter scan, they are probably falsely identified as AGPs. Apart from the PF04674 domain, which seems like an outlier in our analysis, other domains associated with AG motif spans in centaury sequences (Figure 2C) were previously shown to associate with AGPs based on bioinformatics [6,12,13] or experimental evidence [42,44].

### 3.2. Phylogenetic Relations and Structural Features of the Selected FLA, KLA and AG Peptide Sequences

From the identified AGP sequences, we chose 18 transcripts, six from each of the three groups, FLA, KLA and AGp, to analyze their phylogenetic relations and to investigate their response to mechanical wounding, as well as expression in different organs and during morphogenesis in vitro. Even though FLAs are an extensively studied family of putative cell adhesion proteins [19,35,42,45,46], they were of interest for further investigation because their representatives were previously implicated in morphogenesis in vitro in centaury [10]. Most centaury FLAs, just like homologs from other species, are characterized with only one or two fasciclin domains (PF02469) surrounded with AG spans, and most are GPI-anchored proteins (Figure 3). The C-terminal TM region in FLAs is a characteristic of pre-proteins, because the GPI signal sequences contain a stretch of hydrophobic amino acids with properties of a typical transmembrane α-helix, which transiently anchor the pre-proteins to the luminal side of the ER membrane until transamidation with GPI occurs [47,48].

The KLA genes were studied because AG motifs have been only recently linked to a broad range of plant receptor kinases [12,13,43,49], compelling the search for possible roles of this group of proteins. The centaury’s KLAs, as well as related sequences from other plant species, are generally composed of extracellular AG motifs, a transmembrane domain (absent only in CeKLA5) and an intracellular protein kinase domain (Figure 4), as described in detail in Section 2.3. Previously, we have shown that protein kinase (PF00069.25) and protein tyrosine kinase (PF007714.17) are the most frequent Pfam domains found in protein sequences with AG motif spans using the relaxed ragp scan from the 62 analyzed plant proteomes [13]. Since then, an update to the Pfam database has been released, which changed the annotation of PF007714 from “protein tyrosine kinase” (PF007714.17) to “protein tyrosine and serine/threonine kinase” (version PF007714.18). This is in concordance with our own investigation, which indicates that the majority of centaury kinases having AG motif spans with predicted hydroxyprolines are homologous with serine/threonine kinases from other plants (Figure 4). Centaury sequences CeKLA1, CeKLA6 and CeKLA7, along with other members of the clusters C, D and E (Figure 4), are leucine-rich repeats receptor-like kinase (LRR-RLK) chimeric AGPs—molecules implicated in protein-protein interactions and signal transduction [12]. LRR is a domain frequently found in AGPs and HRGPs (Figure 2B,C) which may facilitate dimerization of the molecules that contain it [12]. CeKLA3 and its homologs from the A1 subcluster (Figure 4) belong to the cysteine-rich receptor-like kinase (CRK) family [50,51], characterized by N-terminal salt stress response/antifungal PF01657 domains with conserved cysteines involved in disulfide bridges.

AG peptides, as a subgroup of short classical AGPs, usually do not share sequence homology; nevertheless, one AG peptide, CeAGP3, was identified in an earlier version of *C. erythraea* transcriptome [52] based on a homology search, because it had a conserved domain of unknown function, DUF1070 [10]. We have shown that DUF1070 represents a typical GPI signal sequence associated with few AG motifs [9]; thus, the domains annotation was changed to arabinogalactan peptide PF06376. To the best of our knowledge, PF06376 is the only conserved domain exclusively found in AGPs and HRGPs, in general. Along with CeAGP3, CeAGP7 and CeAGP9, which contain the PF06376 domain, one GPI-anchored peptide without the PF06376 domain (CeAGP6) as well as two partial sequences (CeAGP8 and CeAGP10) were also selected for further study (Figure 5).

The majority of the studied centaury FLA and KLA sequences clustered together with homologues from the taxonomically closest *C. canephora*, emphasizing the interconnection among sequence phylogeny within these protein families and taxonomic relations. This is not surprising since both of these groups contain long and relatively conserved domains with structurally and functionally constrained evolution rates. On the other hand, the AGp phylogenetic tree does not show such trends, with only one reliable cluster (A, Figure 5) from sequences containing the conserved PF06376 domain.

### 3.3. Mechanical Wounding Induces AG Peptides CeAGP6 and CeAGP7 and Downregulates Some FLAs and KLAs

The impact of mechanical wounding was not studied because *C. erythraea* is particularly sensitive to damage caused by natural factors such as strong wind, but because wounding is a common part of plant tissue manipulations in vitro that certainly affects the morphogenic processes [31,32,33]. In this context it was of interest to see whether some AGPs are implicated both in early wounding response and later in somatic embryogenesis and/or organogenesis.

Several lines of evidence suggest that specific AGPs are involved in response to wounding in different plant species. For example, both mRNA and protein levels of two tomato AGPs, a Lys-rich *LeAGP1* and a classical *SlAGP4*, were rapidly up-regulated in response to mechanical wounding in tomato fruits [22]. In centaury, two AG peptides were highly induced by mechanical wounding—*CeAGP6* and *CeAGP7* (Figure 6). *CeAGP6* encodes a 64 amino acid long pro-peptide, which is, after removal of N-sp and GPI signal sequences, reduced to 16 amino acids, with four predicted hydroxyprolines in AG-motif context (27-AOfeafAOAOAOTaes-42). CeAGP7 is a 74 amino acid, PF06376 domain containing, GPI-anchored peptide (Figure 5); the mature protein after processing is only 12 amino acids long with 3 predicted hydroxyprolines in AG-motif context (33-qAOAOAOAatsd-44). CeAGP6 shows similarity to the Arabidopsis At1g55330 (AGP21, Figure 5), while CeAGP7 is similar to Arabidopsis At5g24105 (AGP41, Figure 5). The biological functions of individual arabinogalactan peptides are generally not defined, so translating knowledge in order to provide some context to the observed wounding-induced expression is problematic. One of the sparse reports indicates AtAGP21 is involved in normal root hair formation in Arabidopsis, and that the lack of AtAGP21 at the cell surface stimulates ectopic root hair development similar to that observed in brassinosteroid (BR) mutants [53]. AtAGP21 might act by modifying the responsiveness of the BRI1-BAK1 co-receptor kinase pair to BR or by crosstalk with the BR transduction pathway via thus far unidentified cell surface proteins [53]. Nevertheless, it is apparent that specific membrane-anchored AG peptides are able to transduce signals to the cell and interact with phytohormone signaling pathways. Since the wound-induced signaling involves complex crosstalk among jasmonic acid, ethylene, salicylic acid and abscisic acid [54], as well as BRs [55] signaling pathways, additional investigation needs to be conducted in order to propose a potential physiological significance of CeAGP6 and CeAGP7 induction by wounding.

Finally, there appears to be a causally ambiguous connection between wounding responses and downregulation or inactivation of (certain) AGPs. Namely, in our system, more AGPs are downregulated than upregulated by wounding, including *CeKLA1*, *CeKLA2*, *CeKLA3*, *CeFLA3* and *CeFLA6*, even though this is not statistically significant in all cases (Figure 6). On the other hand, a treatment of Arabidopsis cell suspension culture with βGlcY caused wound-like responses in this system [56]. The responses to βGlcY included the induction of cell wall apposition and callose synthesis, along with changes in gene expression, recorded using a whole-genome microarray, similar to changes caused by wounding [56].

### 3.4. Expression Profiles of AGPs in Different Plant Organs and During Somatic Embryogenesis and Organogenesis in Vitro

The involvement of AGPs in SE and SO from centaury root and leaf explants has been well documented using an array of techniques based on βGlcY applications [10,26,27,28], monoclonal antibodies [29] and expressional analysis [10]. The latter research, however, was based on an in-house transcriptome version of questionable coverage [52] and limited homology-based AGP mining, so only four AGP sequences have been analyzed. Therefore, having a high-quality transcriptome [34] and a powerful tool for AGP mining [13], we had to limit the study to 18 AGPs and to 16 representative centaury organs or developmental stages (Table 1). The set of 16 tissue samples previously used for selection of stable housekeeping genes in centaury [34] turned out to be quite suitable for AGP profiling as well. Namely, having both plants grown in nature and in vitro, samples representing the processes of SE and SO, explants regenerating on hormone-free medium and in the presence of growth regulators, samples cultivated under different light conditions etc., (Table 1) allows for at least coarse discrimination of the effects of in vitro culture, growth regulators or developmental stages on AGPs expression (Figure 7).

A general overview of AGP expression profiles suggests that, in comparison to leaves of plants at a rosette stage grown in vitro (rl) used as a referent sample, other samples from in vitro culture, particularly roots of rosette plants (rr) and whole seedlings (sd), had higher expression of all AG peptides, particularly *CeAGP10*, but comparable levels of FLA and KLA transcripts (Figure 7).

Several AGPs, including *CeAGP8*, *CeAGP10*, *CeFLA3* and *CeKLA3* are more expressed under in vitro conditions (samples rl, rr, sd and rc) than in plants from nature (samples ln, rn, st, mf and imf, Table 1 and Figure 7). On the contrary, FLA transcripts *CeFLA1* and *CeFLA4* were the most abundant in samples from nature, particularly in stems (st). Knowing that stems of centaury in nature are very wiry, it can be speculated that these FLAs, as cell adhesion molecules, may contribute to the stem sturdiness. Indeed, two Arabidopsis FLAs, *AtFLA11* and *AtFLA12*, with quite similar architecture to *CeFL*A1 and *CeFLA4*, having a single FAS domain surrounded by AG motif spans (Figure 2), are important for stem biomechanical properties [57]. It is proposed that FLAs with such architecture contribute to the stem strength by affecting cellulose deposition and to the stem elasticity by affecting the integrity of the cell-wall matrix [57].

It should be noted that *CeFLA1* and *CeFLA4* share not only similar expression profiles in different tissue samples (Figure 7) and in response to wounding (Figure 6), but are also structurally/phylogenetically close (Figure 3). It is not surprising that these two genes had the highest correlation of expression of all gene pairs (Figure 8). *CeFLA1* and *CeFLA4* are also among the few genes with relatively high expression in embryogenic calli, suggesting their potential role in the initiation of SE. *CeFLA1* (previously named *CeAGP1*, GenBank: KC733882, [10]) was highly expressed in centaury leaf explants after 10 days of culture on medium containing 2,4-D and CPPU, when indirect SE occurs [10]. Since *CeFLA1* was expressed at a similar level in leaf explants regenerating both on light and in darkness, it was concluded that it was specifically involved in embryogenesis, and not in organogenesis, because organogenesis does not occur in darkness in this system [10]. The fact that *CeFL*A1 does not have higher expression in samples representing organogenesis as compared to rl sample (Figure 7) only confirms this conclusion. The only other gene upregulated during induction of SE is *CeAGP10*, which is also highly expressed in globular and cotyledonary somatic embryos (gse and cse), but also in other organs from plants cultivated in vitro (Figure 7). The importance of AGPs during somatic embryogenesis in centaury has been well documented [10,29] and recently reviewed [26], and the fact that there is a dynamic change of AGP epitopes during the development of somatic embryos [29] suggests that there are probably more AGPs (or their glycoforms) involved in this process than we have detected so far.

Regarding the process of SO in vitro, none of the selected 18 genes are upregulated in organogenic calli (oc) or in adventitious buds of any origin (ablh, abl and abr), as compared to the rl sample (Figure 7). However, *CeAGP7*, *CeAGP9* and, to a lesser extent, *CeAGP6* and *CeKLA3* are downregulated in several SO-related samples. *CeAGP3* was previously found to be induced during direct root development (in darkness) and direct shoot development (on light) from centaury leaf explants on hormone-free medium, as well as in explants cultivated on 2,4-D and CPPU-containing medium on light, where indirect organogenesis and somatic embryogenesis occur simultaneously [10]. However, the two experimental systems are somewhat different, particularly because in the previous work [10] whole leaf explants with developing adventitious buds and/or somatic embryos were used, whereas here, organogenic calli and adventitious buds were separated from the explants for the RNA extraction; thus, it would be unreliable to compare the obtained results. The importance of AGPs during organogenesis in centaury has been confirmed in different experimental systems via incorporation of βGlcY in the culture medium [10,27,28] and visualization of dynamic changes of AGPs using a set of fluorescent anti-AGP antibodies [29]. The involvement of AGPs in the process of organogenesis has also been confirmed in other species, for example, in wheat [58], sugar beet [59] and grapefruit [60]. Hence, it is very likely that the selected set of 18 AGPs simply did not include those AGPs that are upregulated during regeneration via organogenesis in vitro.

*CeAGP6* and *CeAGP7*, which were involved in immediate response to wounding, with a peak of expression 6 h upon wounding (Figure 6), are downregulated during regeneration via organogenesis and somatic embryogenesis and so can hardly be considered as a link connecting these processes. On the contrary, *CeFLA1*, which is slightly induced upon wounding, but with a clear increasing trend (Figure 6), could be involved in post-wounding events such as healing. The same is true for *CeFLA4*, even though its increase of expression was not statistically significant. Since CeFLA1 and *CeFLA4* are induced in embryogenic calli (Figure 7), they might be a part of the network connecting wounding and somatic embryogenesis. While *CeFLA1* and *CeFLA4* are putatively involved in wounding or healing responses, induction of somatic embryogenesis and stem reinforcement, several AGPs did not significantly change their expression regardless of the treatment or sample type. These include *CeKLA6* and *CeKLA7*—genes that are constitutively expressed in all samples (less than 2 log2 fold changed in any sample compared to rl control) and should be considered for evaluation as potentially good housekeeping genes.

## 4. Materials and Methods

### 4.1. Plant Material

Stock shoot culture of *C. erythraea* was initiated from seeds purchased from Jelitto Staudensamen GmbH, Schwarmstedt, Germany, as previously described by [25]. Three-week-old seedlings were transferred to fresh ½MS [61] medium for further growth. Samples for the wounding experiment were obtained by cutting young rosette leaves from three-month-old plants at the rosette stage 5 mm from the top. Explants were transferred to fresh ½MS medium and sampled after 30 min, 3 h, 6 h, 12 h, 24 h and 48 h. As control, immediately frozen cut leaf tissue was used (0 min).

*C. erythraea* tissue samples from 16 different developmental stages and organs as described by [34] were used (Table 1) to investigate AGP expression.

### 4.2. HRGP Sequence Identification

Filtering *C. erythraea* HRGP sequences was performed using ragp 0.3.2 R package [13]. In silico translated protein sequences from the de novo assembled centaury transcriptome [34] were used as the starting point for HRGP filtering. The protein sequences were first evaluated for the presence of secretory signals, where a majority vote between the predictions from TargetP1.1 [62], SignalP4.1 [63] and Phobius1.01 [64] web servers was used to determine if the sequences contained an N-sp. Sequences predicted to be secreted were used for the prediction of hydroxyproline positions, and sequences containing three or more probable hydroxyprolines were further analyzed. In order to identify and describe prototypical HRGP sequences, the motif and amino acid bias (MAAB) classification [4] as implemented in ragp was used. To resolve ambiguities in MAAB classes which require information about GPI presence, NetGPI1.1 [65] was utilized. For identification of potential arabinogalactan sequences, we performed a relaxed sequence search for clusters of at least three AG glycomodules, no more than 10 amino acids apart as described in [13], and a more stringent search for clusters of least four AG glycomodules, no more than four amino acids apart. Only AG glycomodules with probable hydroxyprolines were considered. GPI prediction in analyzed sequences was performed using NetGPI 1.1 [65], and disorder prediction was performed via ESpritz [66] using the model trained with “X-Ray”train set and default parameters. Domain prediction was performed with hmmscan 3.3.2 [67] using Pfam-A33 database (http://ftp.ebi.ac.uk/pub/databases/Pfam/releases/Pfam33.0, accessed on 12 August 2021). All of the mentioned web servers were queried via the ragp package interface. After filtering, 18 sequences identified as AGP were chosen for expression evaluation based on their primary structure and the ability to construct discriminatory primers: 6 containing fasciclin domains (fasciclin-like AGPs (FLA)), 6 containing protein kinase domains (kinase like AGPs (KLA)) and 6 short AG peptide-like sequences.

### 4.3. RNA Isolation and qPCR

Total RNA from wounded tissue was isolated using CTAB reagent [68]. RNA from all other samples was isolated using TRIzol reagent (Invitrogen—Thermo Fisher Scientific, Waltham, MA, USA) according to the manufacturer’s instructions. The isolations for gene expression studies were performed from three independent biological replicates. RNA was quantified spectrophotometrically using Nano Photometer N60 (Implen) and assessed via agarose gel electrophoresis. Total RNA was treated with DNAse I (Thermo Scientific, Waltham, MA, USA) and reverse transcribed with poly(dT)18 primers, using a RevertAid First Strand cDNA Synthesis Kit (Thermo Scientific, Waltham, MA, USA). Specific primers were designed for the 18 chosen sequences using Primer Blast (www.ncbi.nlm.nih.gov/tools/primer-blast, accessed on 12 August 2021, Supplementary Table S3) and checked for specificity using the de novo assembled centaury transcriptome [34]. The specificity of the primers was confirmed via PCR followed by gel electrophoresis. The obtained amplicons were used for preparation of standards for evaluation of qPCR efficiency. The amplicons were extracted from the gel using a GeneJET Gel Extraction Kit (Thermo Fisher Scientific, Waltham, MA, USA), quantified spectrophotometrically and serially diluted in a 10^9^–10^2^ copies µL^−1^ range. The qPCR reactions were set with Maxima SYBR Green/ROX qPCR Master Mix (2X) (Thermo Fisher Scientific, Waltham, MA, USA), with 0.3 µM specific primers and cDNA corresponding to 12.5 ng RNA in 10 µL total volume. Each reaction was performed in 3 biological replicates. The cycling was performed in the QuantStudio 3 RealTime PCR System (Thermo Fisher Scientific, Waltham, MA, USA). The cycling program included initial denaturation (95 °C/10 min), followed by 40 cycles of denaturation (95 °C/15 s), annealing (at gene-specific Ta/30 s, Supplementary Table S3) and extension (72 °C/30 s). AGP expression data was analyzed using the ΔΔCt method [69]. For normalization of qPCR data, the arithmetic average of Ct values for Ribosomal protein L2 (*RPL2*) and TATA binding protein 1 (*TBP1*) genes, previously shown to be the most stable housekeeping genes in all sample sets by [34], were used.

### 4.4. Phylogenetic Analysis

Phylogeny of the analyzed *C. erythraea* AGP sequences was assessed via comparison with homologs from 18 plant species: *Arabidopsis thaliana*, *Beta vulgaris*, *Capsicum annuum*, *Coffea canephora*, *Cynara cardunculus*, *Daucus carota*, *Glycine max*, *Helianthus annuus*, *Hordeum vulgare*, *Ipomoea triloba*, *Nicotiana attenuata*, *Olea europaea var. sylvestris*, *Oryza sativa Japonica*, *Populus trichocarpa*, *Prunus avium*, *Solanum lycopersicum*, *Triticum aestivum and Zea mays*. The plant species subset contained a mix of well-studied model plants, commercially valuable plants, as well as plants taxonomically related to *C. erythraea*. Proteomes from these species were obtained from Ensembl release 49 (https://plants.ensembl.org/, http://ftp.ensemblgenomes.org/vol1/pub/plants/release-49/fasta, accessed on 12 August 2021). For fasciclin and kinase phylogenies the sequences with Pfam domains PF02469 or PF07714 were queried from the mentioned proteomes using biomartr [70], and protein blast was performed versus chosen *C. erythraea* sequences. Raw bit-score top hits per *C. erythraea* sequence per plant were filtered and the top five homologs per *C. erythraea* sequence were used for phylogeny construction. Sequence alignment was performed using the DECIPHER 2.18.1 R package [71] using default arguments with 10 iterations and 10 refinements. Several models of amino acid replacement—JTT [72], WAG [73], LG [74], Dayhoff [75] and Blosum62 [76]—were tested, and the Bayesian information criterion was used to select the best-fit model. The resulting alignment and best-fit model were used to estimate a sequence distance matrix, which was used to create a neighbor joining (NJ) tree via phangorn R package 2.5.5 [77]. This NJ tree was used as the basis for fitting a maximum likelihood tree with optimization of the gamma rate parameter and proportion of invariable sites using stochastic rearrangement. To assess cluster stability, non-parametric bootstrap was performed for 100 iterations. Phylogeny of AG peptides was assessed using an alignment-free approach. The ragp pipeline was performed on protein sequences with a length of 30–100 amino acids from the 18 plant species. The identified AG peptides were used, along with *C. erythraea* sequences, to estimate a sequence distance matrix using the kmer [78] R package with kmer length 3 and other default parameters. The closest five sequences per *C. erythraea* AG peptide sequence per plant were filtered and the top five homologues per *C. erythraea* AG peptide sequence were used for phylogeny construction, which was performed using neighbor joining on a distance matrix created using the kmer R package with kmer length 3. As with the alignment-based method non-parametric bootstrap was performed for 100 iterations to assess cluster stability. Phylogenetic three clusters with less than 50/100 bootstrap support were collapsed into multichotomies.

### 4.5. Statistical Analyses

Statistical analysis was performed using R programming language for statistical computing [79]. Gene relative expression values from the wounding experiment were analyzed using Student’s *t*-test, where each time slice was compared with the control (0 min). *p*-values obtained from these pairwise comparisons were adjusted using the Benjamini–Hochberg method [80] using all performed comparisons (for all genes). Relative expression values from various centaury tissue and organ samples were analyzed using Welch’s *t*-test, where each sample was compared with the control (rosette leaf-rl). *p*-values obtained from these pairwise comparisons were adjusted using the Benjamini–Hochberg method [80] using all performed comparisons (for all genes). The extent of the linear relationship among the gene expression patterns was estimated using Pearson correlation (cor_Pear_), and the correlation matrix is presented as a heatmap using gplots [81] R package. The rows and columns of the heatmap are arranged according to hierarchical cluster analysis performed on a matrix of correlation distances (1-cor_Pear_, so that maximal positive correlation of 1 becomes 0 distance and maximal negative correlation of −1 becomes distance 2). Cluster agglomeration was performed using the complete linkage method as implemented in R package stats [79]. Mutual dependence of genes based on expression patterns was estimated using the Jackknife bias corrected mutual information pairwise matrix obtained via the R package mpmi [82] with all default parameters.

## 5. Conclusions

The identified centaury HRGP sequences and MAAB class distribution should be considered as preliminary, but for the eventual refinement of the centaury HRGP/AGP family description, the whole genome sequencing, de novo assembly and annotation is required. Having a complete genome would certainly help in completing currently C-truncated sequences such are *CeAGP8* and *CeAGP10* and their proper classification and identification of novel sequences not present in the available transcriptome, as well as identifying regulatory elements of AGPs with interesting expression profiles. Our results confirm that wounding causes changes in AGPs’ expression profiles, but a more systematic approach is required to clarify the role of AGPs such as *CeAGP6* and *CeAGP7* in wound and healing responses. Understanding what players are involved in this process, as well as their roles, can have significant impact not only for fundamental understanding of plant defense, healing and regeneration, but might also be exploited for the improvement of crop tolerance to wind, flooding and insect or herbivore-caused mechanical wounding. While we have identified AGPs with apparently multiple roles, such are *CeFLA1* and *CeFLA4*, other AGPs are likely to be of even greater importance since they are constitutively expressed and thus are candidates for housekeeping genes, such as *CeKLA6* and *CeKLA7*. However, a different selection of genes for testing is required for identification of AGPs specifically involved in embryogenesis and organogenesis in vitro.

## Figures and Tables

**Table 1:** *C. erythraea* tissue samples used for AGP expression analysis. 2,4-D-2,4-dichlorophenoxyacetic acid; CPPU-N-(2-chloro-4pyridyl)-N′-phenylurea.

	Sample	Treatment	Light Conditions	Abbreviation
**Plants and roots** **grown in vitro**	rosette leaves	Solid hormone-free medium	16 h light 8 h darkness	rl
	rosette roots			rr
	seedlings			sd
	root cultures			rc
**Flowering plants** **from nature**	leaves	none	natural	ln
	roots			rn
	stems			st
	mature flowers			mf
	immature flowers			imf
**Organogenesis**	organogenic callus	0.2 mgl^−1^ 2,4-D 0.5 mgl^−1^ CPPU	16 h light 8 h darkness	oc
	adventitious buds formed on leaf explants			ablh
	adventitious buds formed on leaf explants	hormone-free medium		abl
	adventitious buds formed on root explants			abr
**Somatic embryogenesis**	embryogenic callus	0.2 mgl^−1^ 2,4-D 0.5 mgl^−1^ CPPU	darkness	ec
	globular embryos			gse
	cotyledonary embryos			cse

## Data Availability

*C. erythraea* transcriptome and annotation is provided at https://zenodo.org/record/3591805. Identified AGPs are provided in Supplementary Tables S1–S3. The 18 AGPs whose expression was analyzed will be available in NCBI upon manuscript acceptance—accessions are provided in Supplementary Table S3.

## Supplementary Materials

The following are available online at https://www.mdpi.com/article/10.3390/plants10091870/s1, Table S1: MAAB classification of centaury HRGPs, Table S2: ragp identified centaury AGPs and Table S3: 18 selected AGPs info.

## References

[1] Deepak S., Shailasree S., Kini R.K., Muck A., Mithöfer A., Shetty S.H. (2010). Hydroxyproline-rich Glycoproteins and Plant Defence. J. Phytopathol..

[2] Hijazi M., Velasquez S.M., Jamet E., Estevez J.M., Albenne C. (2014). An update on post-translational modifications of hydroxyproline-rich glycoproteins: Toward a model highlighting their contribution to plant cell wall architecture. Front. Plant Sci..

[3] Kieliszewski M.J., Lamport D.T.A., Tan L., Cannon M.C. (2010). Hydroxyproline-Rich Glycoproteins: Form and Function. Annu. Plant Rev..

[4] Johnson K.L., Cassin A.M., Lonsdale A., Bacic A., Doblin M.S., Schultz C.J. (2017). A motif and amino acid bias bioinformatics pipeline to identify hydroxyproline-rich glycoproteins. Plant Physiol..

[5] Ellis M., Egelund J., Schultz C.J., Bacic A. (2010). Arabinogalactan-Proteins: Key Regulators at the Cell Surface?. Plant Physiol..

[6] Showalter A.M., Keppler B., Lichtenberg J., Gu D., Welch L.R. (2010). A bioinformatics approach to the identification, classification, and analysis of hydroxyproline-rich glycoproteins. Plant Physiol..

[7] Sun W., Xu J., Yang J., Kieliszewski M.J., Showalter A.M. (2005). The Lysine-rich Arabinogalactan-protein Subfamily in Arabidopsis: Gene Expression, Glycoprotein Purification and Biochemical Characterization. Plant Cell Physiol..

[8] Baldwin T.C., Domingo C., Schindler T., Seetharaman G., Stacey N., Roberts K. (2001). DcAGP1, a secreted arabinogalactan protein, is related to a family of basic proline-rich proteins. Plant Mol. Biol..

[9] Simonović A.D., Dragićević M.B., Bogdanović M.D., Trifunović-Momčilov M.M., Subotić A.R., Todorović S.I. (2016). DUF1070 as a signature domain of a subclass of arabinogalactan peptides. Arch. Biol. Sci..

[10] Simonović A.D., Filipović B.K., Trifunović M.M., Malkov S.N., Milinković V.P., Jevremović S.B., Subotić A.R. (2015). Plant regeneration in leaf culture of *Centaurium erythraea* Rafn. Part 2: The role of arabinogalactan proteins. Plant Cell Tissue Organ Cult..

[11] Showalter A.M., Keppler B.D., Liu X., Lichtenberg J., Welch L.R. (2016). Bioinformatic Identification and Analysis of Hydroxyproline-Rich Glycoproteins in *Populus trichocarpa*. BMC Plant Biol..

[12] Ma Y., Yan C., Li H., Wu W., Liu Y., Wang Y., Chen Q., Ma H. (2017). Bioinformatics Prediction and Evolution Analysis of Arabinogalactan Proteins in the Plant Kingdom. Front. Plant Sci..

[13] Dragićević M.B., Paunović D.M., Bogdanović M.D., Todorović S.I., Simonović A.D. (2020). ragp: Pipeline for mining of plant hydroxyproline-rich glycoproteins with implementation in R. Glycobiology.

[14] Seifert G.J., Roberts K. (2007). The Biology of Arabinogalactan Proteins. Annu. Rev. Plant Biol..

[15] Majewska-Sawka A., Nothnagel E.A. (2000). The Multiple Roles of Arabinogalactan Proteins in Plant Development. Plant Physiol..

[16] Pereira A.M., Pereira L.G., Coimbra S. (2015). Arabinogalactan proteins: Rising attention from plant biologists. Plant Reprod..

[17] Costa M., Pereira A.M., Pinto S.C., Silva J., Pereira L.G., Coimbra S. (2019). In silico and expression analyses of fasciclin-like arabinogalactan proteins reveal functional conservation during embryo and seed development. Plant Reprod..

[18] Lamport D., Kieliszewski M., Showalter A. (2006). Salt stress upregulates periplasmic arabinogalactan proteins: Using salt stress to analyse AGP function. New Phytol..

[19] Meng J., Hu B., Yi G., Li X., Chen H., Wang Y., Yuan W., Xing Y., Sheng Q., Su Z. (2020). Genome-wide analyses of banana fasciclin-like AGP genes and their differential expression under low-temperature stress in chilling sensitive and tolerant cultivars. Plant Cell Rep..

[20] Gilson P., Gaspar Y.M., Oxley D., Youl J.J., Bacic A. (2001). NaAGP4 is an arabinogalactan protein whose expression is suppressed by wounding and fungal infection in Nicotiana alata. Protoplasma.

[21] Liu C., Mehdy M.C. (2007). A Nonclassical Arabinogalactan Protein Gene Highly Expressed in Vascular Tissues, AGP31, Is Transcriptionally Repressed by Methyl Jasmonic Acid in Arabidopsis. Plant Physiol..

[22] Fragkostefanakis S., Dandachi F., Kalaitzis P. (2012). Expression of arabinogalactan proteins during tomato fruit ripening and in response to mechanical wounding, hypoxia and anoxia. Plant Physiol. Biochem..

[23] Nguema-Ona E., Vicré-Gibouin M., Cannesan M.A., Driouich A. (2013). Arabinogalactan proteins in root-microbe interactions. Trends Plant Sci..

[24] Šiler B., Živković S., Banjanac T., Cvetković J., Nestorović Živković J., Ćirić A., Soković M., Mišić D. (2014). Centauries as underestimated food additives: Antioxidant and antimicrobial potential. Food Chem..

[25] Filipović B.K., Simonović A.D., Trifunović M.M., Dmitrović S.S., Savić J.M., Jevremović S.B., Subotić A.R. (2015). Plant regeneration in leaf culture of *Centaurium erythraea* Rafn. Part 1: The role of antioxidant enzymes. Plant Cell Tissue Organ Cult..

[26] Simonović A.D., Trifunović-Momčilov M.M., Filipović B.K., Marković M.P., Bogdanović M.D., Subotić A.R. (2021). Somatic Embryogenesis in *Centaurium erythraea* Rafn—Current Status and Perspectives: A Review. Plants.

[27] Trifunović M., Tadić V., Petrić M., Jontulović D., Jevremović S., Subotić A. (2014). Quantification of arabinogalactan proteins during in vitro morphogenesis induced by β-d-glucosyl Yariv reagent in *Centaurium erythraea* root culture. Acta Physiol. Plant..

[28] Trifunović M., Subotić A., Petrić M., Jevremović S., Rybczyński J.J., Davey M.R., Mikuła A. (2015). The Role of Arabinogalactan Proteins in Morphogenesis of *Centaurium erythraea* Rafn In Vitro. The Gentianaceae—Volume 2: Biotechnology and Applications.

[29] Filipović B.K., Trifunović-Momčilov M.M., Simonović A.D., Jevremović S.B., Milošević S.M., Subotić A.R. (2021). Immunolocalization of some arabinogalactan protein epitopes during indirect somatic embryogenesis and shoot organogenesis in leaf culture of centaury (*Centaurium erythraea* Rafn). In Vitro Cell. Dev. Biol. Plant.

[30] Lup S.D., Tian X., Xu J., Pérez-Pérez J.M. (2016). Wound signaling of regenerative cell reprogramming. Plant Sci..

[31] Méndez-Hernández H.A., Ledezma-Rodríguez M., Avilez-Montalvo R.N., Juárez-Gómez Y.L., Skeete A., Avilez-Montalvo J., De-la-Peña C., Loyola-Vargas V.M. (2019). Signaling Overview of Plant Somatic Embryogenesis. Front. Plant Sci..

[32] Santarem E.R., Pelissier B., Finer J.J. (1997). Effect of explant orientation, pH, solidifying agent and wounding on initiation of soybean somatic embryos. In Vitro Cell. Dev. Biol. Plant.

[33] Bhatia P., Ashwath N., Midmore D.J. (2005). Effects of genotype, explant orientation, and wounding on shoot regeneration in tomato. In Vitro Cell. Dev. Biol. Plant.

[34] Ćuković K., Dragićević M., Bogdanović M., Paunović D., Giurato G., Filipović B., Subotić A., Todorović S., Simonović A. (2020). Plant regeneration in leaf culture of *Centaurium erythraea* Rafn. Part 3: De novo transcriptome assembly and validation of housekeeping genes for studies of in vitro morphogenesis. Plant Cell Tissue Organ Cult..

[35] He J., Zhao H., Cheng Z., Ke Y., Liu J., Ma H. (2019). Evolution Analysis of the Fasciclin-Like Arabinogalactan Proteins in Plants Shows Variable Fasciclin-AGP Domain Constitutions. Int. J. Mol. Sci..

[36] Simão F.A., Waterhouse R.M., Ioannidis P., Kriventseva E.V., Zdobnov E.M. (2015). BUSCO: Assessing genome assembly and annotation completeness with single-copy orthologs. Bioinformatics.

[37] Yang Y., Smith S.A. (2013). Optimizing *de novo* assembly of short-read RNA-seq data for phylogenomics. BMC Genom..

[38] Gruenheit N., Deusch O., Esser C., Becker M., Voelckel C., Lockhart P. (2012). Cutoffs and k-mers: Implications from a transcriptome study in allopolyploid plants. BMC Genom..

[39] Ono H., Ishii K., Kozaki T., Ogiwara I., Kanekatsu M., Yamada T. (2015). Removal of redundant contigs from *de novo* RNA-Seq assemblies via homology search improves accurate detection of differentially expressed genes. BMC Genom..

[40] Kerkvliet J., de Fouchier A., van Wijk M., Groot A.T. (2019). The Bellerophon pipeline, improving *de novo* transcriptomes and removing chimeras. Ecol. Evol..

[41] Johnson K.L., Cassin A.M., Lonsdale A., Wong G.K.-S., Soltis D.E., Miles N.W., Melkonian M., Melkonian B., Deyholos M.K., Leebens-Mack J. (2017). Insights into the Evolution of Hydroxyproline-Rich Glycoproteins from 1000 Plant Transcriptomes. Plant Physiol..

[42] Johnson K.L., Jones B.J., Bacic A., Schultz C.J. (2003). The fasciclin-like arabinogalactan proteins of Arabidopsis. A multigene family of putative cell adhesion molecules. Plant Physiol..

[43] Pfeifer L., Shafee T., Johnson K.L., Bacic A., Classen B. (2020). Arabinogalactan-proteins of *Zostera marina* L. contain unique glycan structures and provide insight into adaption processes to saline environments. Sci. Rep..

[44] Mashiguchi K., Yamaguchi I., Suzuki Y. (2004). Isolation and Identification of Glycosylphosphatidylinositol-Anchored Arabinogalactan Proteins and Novel β-Glucosyl Yariv-Reactive Proteins from Seeds of Rice (*Oryza sativa*). Plant Cell Physiol..

[45] Seifert G.J. (2018). Fascinating Fasciclins: A Surprisingly Widespread Family of Proteins that Mediate Interactions between the Cell Exterior and the Cell Surface. Int. J. Mol. Sci..

[46] Shafee T., Bacic A., Johnson K. (2020). Evolution of Sequence-Diverse Disordered Regions in a Protein Family: Order within the Chaos. Mol. Biol. Evol..

[47] Kinoshita T., Fujita M. (2016). Thematic Review Series: Glycosylphosphatidylinositol (GPI) Anchors: Biochemistry and Cell Biology Biosynthesis of GPI-anchored proteins: Special emphasis on GPI lipid remodeling. J. Lipid Res..

[48] Galian C., Björkholm P., Bulleid N., von Heijne G. (2012). Efficient Glycosylphosphatidylinositol (GPI) Modification of Membrane Proteins Requires a C-terminal Anchoring Signal of Marginal Hydrophobicity*. J. Biol. Chem..

[49] Hervé C., Siméon A., Jam M., Cassin A., Johnson K.L., Salmeán A.A., Willats W.G.T., Doblin M.S., Bacic A., Kloareg B. (2016). Arabinogalactan proteins have deep roots in eukaryotes: Identification of genes and epitopes in brown algae and their role in *Fucus serratus* embryo development. New Phytol..

[50] Chen Z. (2001). A superfamily of proteins with novel cysteine-rich repeats. Plant Physiol..

[51] Zuo C., Liu H., Lv Q., Chen Z., Tian Y., Mao J., Chu M., Ma Z., An Z., Chen B. (2020). Genome-Wide Analysis of the Apple (*Malus domestica*) Cysteine-Rich Receptor-Like Kinase (CRK) Family: Annotation, Genomic Organization, and Expression Profiles in Response to Fungal Infection. Plant Mol. Biol. Report..

[52] Malkov S.N., Simonović A.D. Shotgun assembly of *Centaurium erythraea* transcriptome. Proceedings of the 19th Symposium of the Serbian Plant Physiology Society, Book of Abstracts.

[53] Borassi C., Gloazzo Dorosz J., Ricardi M.M., Carignani Sardoy M., Pol Fachin L., Marzol E., Mangano S., Rodríguez Garcia D.R., Martínez Pacheco J., Rondón Guerrero Y.D.C. (2020). A cell surface arabinogalactan-peptide influences root hair cell fate. New Phytol..

[54] Savatin D.V., Gramegna G., Modesti V., Cervone F. (2014). Wounding in the plant tissue: The defense of a dangerous passage. Front. Plant Sci..

[55] Saini S., Sharma I., Pati P.K. (2015). Versatile roles of brassinosteroid in plants in the context of its homoeostasis, signaling and crosstalks. Front. Plant Sci..

[56] Guan Y., Nothnagel E.A. (2004). Binding of Arabinogalactan Proteins by Yariv Phenylglycoside Triggers Wound-Like Responses in Arabidopsis Cell Cultures. Plant Physiol..

[57] MacMillan C.P., Mansfield S.D., Stachurski Z.H., Evans R., Southerton S.G. (2010). Fasciclin-like arabinogalactan proteins: Specialization for stem biomechanics and cell wall architecture in Arabidopsis and Eucalyptus. Plant J..

[58] Konieczny R., Swierczyńska J., Czaplicki A.Z., Bohdanowicz J. (2007). Distribution of pectin and arabinogalactan protein epitopes during organogenesis from androgenic callus of wheat. Plant Cell Rep..

[59] Wiśniewska E., Majewska-Sawka A. (2007). Arabinogalactan-proteins stimulate the organogenesis of guard cell protoplasts-derived callus in sugar beet. Plant Cell Rep..

[60] Orbović V., Göllner E.M., Soria P. (2013). The effect of arabinogalactan proteins on regeneration potential of juvenile citrus explants used for genetic transformation by Agrobacterium tumefaciens. Acta Physiol. Plant..

[61] Murashige T., Skoog F. (1962). A Revised Medium for Rapid Growth and Bio Assays with Tobacco Tissue Cultures. Physiol. Plant..

[62] Emanuelsson O., Brunak S., von Heijne G., Nielsen H. (2007). Locating proteins in the cell using TargetP, SignalP and related tools. Nat. Protoc..

[63] Petersen T.N., Brunak S., von Heijne G., Nielsen H. (2011). SignalP 4.0: Discriminating signal peptides from transmembrane regions. Nat. Methods.

[64] Käll L., Krogh A., Sonnhammer E.L. (2007). Advantages of combined transmembrane topology and signal peptide prediction—The Phobius web server. Nucleic Acids Res..

[65] Gíslason M.H., Nielsen H., Almagro Armenteros J.J., Johansen A.R. (2021). Prediction of GPI-anchored proteins with pointer neural networks. Curr. Res. Biotechnol..

[66] Walsh I., Martin A.J.M., Di Domenico T., Tosatto S.C.E. (2011). ESpritz: Accurate and fast prediction of protein disorder. Bioinformatics.

[67] Eddy S.R. (2011). Accelerated Profile HMM Searches. PLoS Comput. Biol..

[68] Gašić K., Hernandez A., Korban S.S. (2004). RNA extraction from different apple tissues rich in polyphenols and polysaccharides for cDNA library construction. Plant Mol. Biol. Report..

[69] Livak K.J., Schmittgen T.D. (2001). Analysis of relative gene expression data using real-time quantitative PCR and the 2(-Delta Delta C(T)) Method. Methods.

[70] Drost H.-G., Paszkowski J. (2017). Biomartr: Genomic data retrieval with R. Bioinformatics.

[71] Wright E.S. (2015). DECIPHER: Harnessing local sequence context to improve protein multiple sequence alignment. BMC Bioinform..

[72] Jones D.T., Taylor W.R., Thornton J.M. (1992). The rapid generation of mutation data matrices from protein sequences. Comput. Appl. Biosci..

[73] Whelan S., Goldman N. (2001). A General Empirical Model of Protein Evolution Derived from Multiple Protein Families Using a Maximum-Likelihood Approach. Mol. Biol. Evol..

[74] Le S.Q., Gascuel O. (2008). An Improved General Amino Acid Replacement Matrix. Mol. Biol. Evol..

[75] Dayhoff M.O., Schwartz R.M., Orcutt B.C., Dayhoff M.O. (1978). A model of evolutionary change in proteins. Atlas of Protein Sequence and Structure.

[76] Henikoff S., Henikoff J.G. (1992). Amino acid substitution matrices from protein blocks. Proc. Natl. Acad. Sci. USA.

[77] Schliep K.P. (2010). phangorn: Phylogenetic analysis in R. Bioinformatics.

[78] Wilkinson S. kmer: An R Package for Fast Alignment-Free Clustering of Biological Sequences; R Package Version 1.1.1. https://CRAN.R-project.org/package=kmer.

[79] R Core Team (2020). R: A Language and Environment for Statistical Computing.

[80] Benjamini Y., Hochberg Y. (1995). Controlling the False Discovery Rate: A Practical and Powerful Approach to Multiple Testing. J. R. Stat. Soc. Ser. B.

[81] Warnes G.R., Bolker B., Bonebakker L., Gentleman R., Huber W., Liaw A., Lumley T., Maechler M., Magnusson A., Moeller S. (2020). gplots: Various R Programming Tools for Plotting Data. R Package Version 3.1.1. https://CRAN.R-project.org/package=gplots.

[82] Pardy C. (2020). mpmi: Mixed-Pair Mutual Information Estimators. R Package Version 0.43.1. https://CRAN.R-project.org/package=mpmi.

